# Scoring and psychometric properties of the Eye-Drop Satisfaction Questionnaire (EDSQ), an instrument to assess satisfaction and compliance with glaucoma treatment

**DOI:** 10.1186/1471-2415-10-1

**Published:** 2010-02-01

**Authors:** Antoine Regnault, Muriel Viala-Danten, Hélène Gilet, Gilles Berdeaux

**Affiliations:** 1 Mapi Values, Lyon, France; 2 Alcon France, Rueil-Malmaison, France

## Abstract

**Background:**

The objective of this study was to ascertain the scoring and assess the psychometric properties of the Eye-Drop Satisfaction Questionnaire (EDSQ), a 43-item Patient-Reported Outcome instrument developed to assess patients' satisfaction and compliance with glaucoma treatment.

**Methods:**

The EDSQ was administered during an observational, retrospective study to 184 French patients treated for glaucoma. The hypothesized structure, including six dimensions (patient-clinician relationship; patient experience; patient-treatment interaction; apprehension; patient knowledge; travel), was tested by assessing the internal consistency reliability (Cronbach's alpha) and construct-related validity (item convergent and discriminant validity). As unsatisfactory results were demonstrated, another structure was defined using a principal component analysis (PCA) combined with content of items. Psychometric properties of this new structure were assessed. Scores were compared between low, moderate and high compliance profile groups defined using data collected with the Travalert electronic device.

**Results:**

Analyses were performed with the 169 patients who completed at least half of the EDSQ items. The hypothesized structure showed a Cronbach's alpha lower than 0.70 for four dimensions out of six and an overall poor construct-related validity (range of item-scale correlations: 0.00-0.68). The new structure obtained with the PCA included six dimensions: concern about treatment (five items); concern about disease (two items); satisfaction with patient-clinician relationship (five items); positive beliefs (three items); treatment convenience (three items); and self-declared compliance (three items). A score ranging from 0 to 100 was calculated for each dimension, with higher scores indicating more of the attribute referred to in the dimension. Internal consistency reliability was good (Cronbach's alpha greater than 0.70 for five dimensions). The structure offered good construct-related validity (range of item-scale correlations: 0.36-0.82). Ceiling effects of 21% and 49%, were observed for the satisfaction with patient-clinician relationship and self-declared compliance scores. Patients in low compliance profile group reported the lowest score for the satisfaction with patient-clinician relationship, positive beliefs, treatment convenience and self-declared compliance dimensions, and the highest score for the concern about treatment dimension.

**Conclusions:**

The scoring of the EDSQ was developed and the questionnaire proved to have satisfactory psychometric properties. EDSQ scores showed a promising relationship to compliance profiles. The EDSQ could be used in future studies.

## Background

Glaucoma is a group of conditions that damage the eye's optic nerve, causing progressive loss of sight. A higher intra-ocular pressure has been found to be associated with an increased risk of glaucoma. Glaucoma is the second most common cause of worldwide blindness after cataracts[1]. The most common type of glaucoma is Primary Open Angle Glaucoma (POAG), with the only noticeable sign being the gradual loss of vision. Treatments of POAG are aimed at reducing the intraocular pressure (IOP) and include mainly topical therapy, even if other treatments such as surgery or laser can be used for the most severe cases. Treatments are used to slow or prevent the disease progression as glaucoma cannot be totally cured and damage caused cannot be reversed[2]. As patients do not experience any symptoms and as POAG is an irreversible condition, the benefit of treatment is not directly perceived by the patient. In addition, patients treated with eye drops are confronted with many constraints, including the lifelong nature of the treatment, the complexity of the treatment regimen (e.g. instillation at set times, several eye-drop intakes per day, difficulty in self-administration of eye-drops), and the risk of side effects such as eye irritation or eye pain. Thus the risk of non-compliance is high[3-6], for example omission of eye-drop intake or voluntary interruption of the treatment over a few days. Since the effectiveness of glaucoma treatment depends on strict compliance, management of patients should take into account the risk of non-compliance. Compliance and its measure is a real issue in the treatment of glaucoma[4] and factors having an impact on compliance are worthwhile to explore. Complex dosing regimens[7] and patients' perception and knowledge about their illness and its treatment[8,9] have been identified as potential barriers to compliance. Continuing such investigation may result in the discovery of ways to improve compliance of patients treated for glaucoma, and therefore to improve the effectiveness of treatments.

According to theoretical satisfaction models, satisfaction of individuals impacts on their subsequent behaviour [10]. This assertion, when translated in the context of the uptake of a treatment, means that the satisfaction with a treatment has an effect on patient compliance and continuation of a treatment. In practice, higher satisfaction with a treatment has been reported to be associated with better treatment compliance in glaucoma[11,12], as well as in other chronic diseases such as asthma[13] or diabetes[14]. Assessing patients' satisfaction with a glaucoma treatment is of particular interest in the context of measurement of patients' compliance with glaucoma treatment.

The Eye-Drop Satisfaction Questionnaire (EDSQ) has been developed to assess patients' satisfaction and compliance with glaucoma treatment. The development of the conceptual framework of the questionnaire (items and dimensions) have been published previously[15]. The objective of this paper is to present the scoring method and the psychometric properties of the EDSQ.

## Methods

### Patients and study design

An observational, multicenter, cross-sectional and retrospective study was conducted in France with glaucoma-specialized ophthalmologists selected from a list of ophthalmologists prescribing the Travalert^CE ^device to their patients. To be eligible for the study, patients had to be over 18 years old and diagnosed with POAG, such as juvenile glaucoma, exfoliative glaucoma, pigmentary glaucoma or normal tension glaucoma, or high IOP. Patients who were using the Travalert device for administration of treatment for at least 6 weeks and who agreed to participate were included in the study. Patients with secondary glaucoma (congenital glaucoma, inflammatory glaucoma, angle-closure or narrow angle glaucoma following cataract surgery), patients taking non ocular treatment to be taken three times per day or more or associated with a strong burden (e.g. anticoagulant), and patients with chronic eye dryness requiring the instillation of more than five drops a day were not included in the study. A total of 184 patients were enrolled in the study.

At the inclusion visit, ophthalmologists had to complete a medical questionnaire for each patient, including information about ocular and non-ocular co-morbidity, non medical treatment of POAG/IOP (surgery or laser), visual acuity and IOP measurement. Ocular co-morbidities included age-related macular degeneration, retinal detachment, non operated cataract, diabetic retinopathy and uveitis; other co-morbidities included 17 common conditions such as myocardial infarction, dementia, diabetes, cancers and AIDS. At this visit, patients were asked to complete the EDSQ.

### The EDSQ

The EDSQ is a self-administered questionnaire that aims to assess patients' satisfaction and compliance with eye-drop treatment for glaucoma or IOP. It was developed simultaneously in French and UK English[15]. Patient and clinician interviews were conducted to ascertain the conceptual model for satisfaction with eye-drop treatment and to collect patients' comments verbatim. Cognitive debriefing was then conducted with French and English patients to pre-test the questionnaire (assessment of questionnaire's clarity, ease of comprehension and cultural equivalence). The EDSQ included 43 items covering six hypothesized dimensions: patient characteristics (14 items) including apprehension (three items) and travel (three items), treatment characteristics (four items), patient-clinician relationship (seven items), patient experience (seven items), patient-treatment interaction (eight items) and patient knowledge (three items). The 43 items had different response scales: one was continuous (age), eight were dichotomous, two were categorical and thirty-two were ordinal, with different response options.

### Indicator of patient compliance

Patient compliance was evaluated using Travalert, an electronic device allowing the actual use of eye drops to be assessed by reminding the patient of the times for instillation and recording information on each eye-drop dispensed. The data collected with Travalert during a recent study conducted with 10 volunteers[16] were shown to be accurate and reproducible in terms of the date and time-stamping mechanisms. However, the device may overestimate patients' compliance as Travalert may count drops that were not actually instilled in the eye.

The data collected with Travalert in the present study were used to categorize patients into three compliance profiles: high compliance, moderate compliance and low compliance. This categorization was performed using statistical clustering methods. The results have been described in more detail elsewhere[17]. Among the 184 patients included in the study, 113 had enough data from the Travalert device to be classified in one of the three compliance profiles.

### Statistical analyses

#### Analysis population

Analyses were performed for all patients with an assessable EDSQ, that is, with at least 50% of the 43 EDSQ items completed. Age groups were defined using terciles resulting from the description of age in the analysis population.

### Scoring of the EDSQ

The quality of completion was analysed on all EDSQs received. The first structure tested was composed of six scores with 27 ordinal items, based on the EDSQ hypothesized structure: patient-clinician relationship (five items), patient experience (seven items), patient-treatment interaction (six items), patient knowledge (three items), apprehension (three items) and travel (three items). The items not included in this structure were not used for the analyses. In particular, the items about patient demographics and the items about treatment characteristics (e.g. number of instillations per day or time of instillation) were not included since a unidimensional concept could not be hypothesized for them. The psychometric properties of the hypothesized structure, including the internal consistency reliability and construct-related validity, were tested. Reliability coefficients of the scores (i.e. ratio of variance in true scores to the variance in observed scores) were estimated using Cronbach's coefficient alpha. Cronbach's alpha assesses internal consistency reliability and is calculated from the correlations among items included in a score and the number of items in the score. It ranges from 0 to 1, with a recommended threshold of 0.70[18]. The construct-related validity was determined by assessing the item convergent validity (the correlation of an item with its own dimension should be higher than 0.40) and item discriminant validity (the correlation of an item with its own dimension should be higher than with all the other dimensions). The percentage at floor, i.e. the percentage of patients reporting the lowest possible score, and the percentage at ceiling, i.e. the percentage of patients reporting the highest possible score were also described. Unsatisfactory results were found for the hypothesized structure. Therefore, a second structure was defined on the same 27 items using a principal component analysis (PCA) with Varimax rotation. An orthogonal rotation was preferred to an oblique rotation (e.g. Promax) because the content variety of EDSQ items entailed the creation of scores as independent as possible. The selection criterion for dimensions was eigenvalues greater than one. Items were retained in the factor for which they had the highest loading greater than 0.40. The PCA was applied to the 145 patients with no missing data for the 27 items included in the analysis. The psychometric properties of this new structure were tested in a similar way as for the hypothesized structure.

The EDSQ scores were calculated using the factor structure resulting from the PCA, with each item contributing equally to the score, and linearly transformed to a 0-100 scale as follows: (mean of item scores - 1) × 25.

### Description of EDSQ scores

The description of the final EDSQ scores was performed on the overall analysis population, as well as by gender and by age group. The EDSQ scores were also described according to the compliance profiles of patients defined from the Travalert classification.

### Statistical tests, level of significance and software

A Mann-Whitney-Wilcoxon test was used to compare a continuous variable between two groups of patients, and a Kruskal-Wallis test was used to compare a continuous variable between more than two groups of patients. The threshold for statistical significance was fixed at 5%. All analyses were performed using SAS^® ^software for Windows (SAS v9.1; SAS Institute, Inc., Cary, NC, USA).

## Results

### Description of patients' characteristics

Among the 184 patients included in the study, 169 completed at least 50% of the EDSQ items and were included in the analysis population. The mean age was 65 years (Table 1). There was a balanced number of males and females (83 and 85, respectively), with most patients being retired and not living alone (61% and 78%, respectively). A minority of patients (11%) had an ocular co-morbidity and 30% had a non-ocular co-morbidity; 17% had had surgery for POAG or IOP and 10% had been treated by laser. At the inclusion visit, the mean IOP was 16.42 mm Hg for the worse eye, and the median visual acuity for the best eye was 10 (decimal). The classification based on data collected with the Travalert device categorized the majority of patients in the "high compliance" group (57%), while 21% were in the "moderate compliance" group and 22% in the "low compliance" group.

**Table 1:** Demographics, clinical characteristics and compliance profile of patients included in the analysis population (N = 169)

		Analysis population (N = 169)	
**Age **(MD = 1)	Mean (STD)	65.1	(11.8)
	Median (range)	67.5	(18-89)

**Gender**, n (%) (MD = 1)	Male	83	(49.1)
	Female	85	(50.3)

**Professional status**, n (%) (MD = 1)	Working full-time	37	(21.9)
	Working part-time	13	(7.7)
	Retired	103	(61.0)
	Unemployed	3	(1.8)
	House	12	(7.1)

**Patient living alone, **n (%) **(MD = 1)**	No	131	(77.5)
	Yes	37	(21.9)

**Comorbidities, **n (%) (MD = 1)	Ocular	19	(11.2)
	Other	50	(29.6)

**Nonmedicated treatment of POAG/IOP, **n (%) (MD = 1)	Surgery	28	(16.6)
	Laser	17	(10.1)

**IOP^a ^(worst eye) **(MD = 5)	Mean (STD)	16.42	(3.87)
	Median (range)	16	(9-40)

**Visual acuity^b ^(best eye, Decimal) (MD = 13)**	Median (range)	10.00	(1-12)

**Compliance profiles**, n (%) (MD = 56)	Low compliance	25	(22.1)
	Moderate compliance	24	(21.2)
	High compliance	64	(56.6)

STD, Standard Deviation; MD, Missing Data; POAG, Primary Open Angle Glaucoma; IOP, Intraocular Pressure

^a^mm Hg; ^b^decimal scale.

### Quality of completion and distribution of item scores

On average 1.3% of the item responses were missing; 79% of patients did not have any missing items. The item with the highest number of missing data was the item about the number of different ophthalmic drops taken daily (4.1%).

For 23 of the 30 EDSQ items with an ordinal response scale, most patients used one of the two most favourable response categories. Indeed, the majority of patients answered the most favourable response choices for the items about their satisfaction (e.g. "very" or "extremely" to satisfaction with care, satisfaction with information, and treatment convenience), their compliance (e.g. "always" to self-declared compliance over the last 4 weeks, "never" to default in instillation of eye drops in general), and the nuisance of treatment (e.g. "not at all" to concern about putting things into one's eyes and blink reflex).

### Ascertaining the scoring of the EDSQ

For the hypothesized structure including 27 items, Cronbach's alphas ranged from 0.38 for the travel dimension to 0.73 for the patient experience dimension (Table 2). All items of the apprehension dimension and almost all items of the patient knowledge dimension met the convergent and discriminant validity criteria. For the patient-clinician relationship, patient experience, patient-treatment interaction and travel dimensions, 80%, 57%, 33% and 67% of the items, respectively, met the convergent validity criterion, and 85%, 79%, 71% and 67% of the items, respectively, met the discriminant validity criterion. A floor effect was observed for the apprehension dimension with 28% of patients reporting the lowest possible score, and a ceiling effect was observed for the patient knowledge dimension with 30% of patients reporting the highest possible score.

**Table 2:** Item convergent and discriminant validity, internal consistency reliability, and floor and ceiling effect obtained using the hypothesized and exploratory structures of the EDSQ - Patients with complete EDSQ data (N = 145)

Dimensions	No. of items	Range of item-scale correlations	Item convergent validity^a ^(% success)	Item discriminant validity^b ^(% success)	Cronbach's alpha	Floor (%)	Ceiling (%)
***Hypothesized structure resulting from the development phase***							

Apprehension	3	0.42-0.54	100.0	100.0	0.66	28.3	0.0
Patient-clinician relationship	5	0.10-0.68	80.0	88.0	0.65	0.0	1.4
Patient experience	7	0.12-0.62	57.1	80.0	0.73	0.7	0.0
Patient knowledge	3	0.42-0.53	100.0	93.3	0.70	0.0	30.3
Patient-treatment interaction	6	0.08-0.51	33.3	76.7	0.56	0.0	6.9
Travel	3	0.00-0.46	66.7	66.7	0.38	5.5	0.0

***Exploratory structure resulting from the PCA***							

Concern about treatment	5	0.45-0.64	100.0	100.0	0.78	18.9	0.0
Concern about disease	2	0.82-0.82	100.0	100.0	0.90	8.8	5.4
Satisfaction with patient-clinician relationship	5	0.49-0.73	100.0	100.0	0.78	0.0	20.9
Positive beliefs	3	0.49-0.67	100.0	93.3	0.74	0.0	4.7
Treatment convenience	3	0.50-0.60	100.0	100.0	0.72	0.7	13.5
Self-declared compliance	3	0.36-0.46	66.7	100.0	0.65	0.0	49.3

PCA, Principal Component Analysis

^a^Success criteria: item-scale correlation greater than 0.4.

^b^For each item, correlation coefficient with its own scale is compared with correlation coefficients with the other scales. Reported is the percentage of all pairwise comparisons where the correlation of an item with its own scale is greater than correlation with the other scale.

The PCA conducted on the same 27 items to define an alternative structure for the EDSQ led to eight factors with eigenvalues greater than one, explaining 65% of the total variance. Among the eight factors, two were not retained because of poor consistency in the content of items included in each of these two factors. Thus the five items included in these two factors were not included in the final structure of the EDSQ. In addition, the item about consultation frequency loaded on a factor with items related to patients' concern about their disease. This content difference led to the exclusion of this item from the final structure. However, these six items were kept in the questionnaire to be analysed separately in future studies. Six dimensions were then selected and named, based on the content of the items: concern about treatment (five items), concern about disease (two items), satisfaction with patient-clinician relationship (five items), positive beliefs (three items), treatment convenience (three items) and self-declared compliance (three items). The Cronbach's alpha of each dimension was higher than 0.70, except for the self-declared compliance dimension (0.65) (Table 2). All items but one ("Frequency of voluntary break" in the self-declared compliance dimension) met the convergent validity criterion and all items but one ("Confidence in the effectiveness of the treatment" in the positive beliefs dimension) met the discriminant validity criterion. A ceiling effect was observed for the satisfaction with patient-clinician relationship and the self-declared compliance dimensions (21% and 49%, respectively, of patients reporting the highest possible score), and a floor effect was observed for the concern about treatment dimension (19% of patients reporting the lowest possible score).

The final structure of the EDSQ included six dimensions based on 21 items (Table 3). A score was calculated for each of the six dimensions as the mean of items in the dimension with all items contributing equally to the score, and was linearly transformed to a 0-100 scale. Some items were reversed so that higher dimension scores reflected more of the attribute referred to in the dimension (e.g. higher concern about disease, greater satisfaction with patient-clinician relationship). Missing items were handled using the half-scale rule (i.e. if at least 50% of the items of the dimension are completed, missing items are replaced by the mean of non-missing items of the dimension; otherwise the dimension score is set to missing).

**Table 3:** Scoring of the EDSQ

Dimension	Included items	Score calculation^a^
Concerns about treatment *(five items)*	Discomfort Worries about putting things in eyes Blink reflex Treatment as a burden Feeling about lifelong treatment	If at least three items are available: (Mean of item scores - 1) × 25

Concerns about disease *(two items)*	Fear of disease evolution Thinking about the disease consequences	If at least one item is available: (Mean of item scores - 1) × 25

Satisfaction with patient-clinician relationship *(five items)*	Satisfaction with visit frequency Satisfaction with the information given by the ophthalmologist Frequency of information given by the ophthalmologist about eye-pressure level Frequency of information given by the ophthalmologist about visual acuity Satisfaction with care	If at least three items are available: (Mean of item scores - 1) × 25

Positive beliefs *(three items)*	Confidence in the effectiveness of the treatment Feeling about feedback and motivation Feeling about follow-up and motivation	If at least two items are available: (Mean of item scores - 1) × 25

Treatment convenience *(three items)*	Convenience of the delivery system in bottle opening Convenience of the delivery system in drop dosing Convenience of storage conditions	If at least two items are available: (Mean of item scores - 1) × 25

Self-declared compliance *(three items)*	Self-assessed compliance over the last 4 weeks Frequency treatment is forgotten in general Frequency of voluntary break from treatment	If at least two items are available: (Mean of item scores - 1) × 25

^a^Higher dimension scores reflect more of the attribute implied by the name (e.g. higher concern about disease, greater satisfaction with patient-clinician relationship).

### EDSQ scores according to demographic parameters (age, gender)

A statistically significant difference in the concern about disease score was observed according to gender (p = 0.002), with a higher score for females (mean of 56.4 vs. 42.5 for males) (Table 4). A statistically significant difference in the treatment experience score was observed according to age group (p = 0.020), with a lower score for patients older than 72 years (mean of 67.7 vs. 73.2 for patients between 60 and 72 years and 77.8 for patients younger than 60 years). The other EDSQ scores did not differ statistically between gender or age groups.

**Table 4:** EDSQ mean dimension scores, overall and according to age and gender (N = 168)

Dimension	Total	Age				Gender		
			
		<60(n = 56)	60-72 (n = 56)	>72 (n = 55)	p-value^a^	Male (n = 83)	Female (n = 85)	p-value^b^
Concern about treatment	21.0	22.1	19.8	20.7	0.785	18.9	22.8	0.197
Concern about disease	49.6	56.5	46.1	45.8	0.082	**42.5**	**56.4**	**0.002**
Satisfaction with patient-clinician relationship	83.9	84.6	84.8	82.5	0.634	86.1	82.0	0.159
Positive beliefs	68.1	67.8	70.6	66.1	0.530	69.5	66.8	0.634
Treatment convenience	72.8	**77.8**	**73.2**	**67.7**	**0.020**	72.1	73.7	0.500
Self-declared compliance	93.0	91.2	93.7	93.8	0.322	92.8	93.0	0.762

^a^Kruskal-Wallis test; ^b^Mann-Whitney-Wilcoxon test.

In bold: statistically significant differences at the 0.05 threshold.

### Relationships between EDSQ scores and compliance profile

No statistically significant differences in the EDSQ scores were observed between the three compliance profile groups (Table 5). The satisfaction with patient-clinician relationship score presented the highest association with compliance (p = 0.079). The lowest satisfaction with patient-clinician relationship score was observed for patients in the low compliance profile group (mean of 77.7 vs. 87.5 for patients in the moderate compliance profile group and 83.8 for patients in the high compliance profile group).

**Table 5:** Association between compliance profiles and EDSQ dimension scores (N = 113; population documented for compliance and EDSQ): Mean scores and p-value using a Kruskal-Wallis test

Dimensions	Compliance profile^a^			
	
	Low compliance (n = 25)	Moderate compliance (n = 24)	High compliance (n = 64)	p-value
Concern about treatment	22.2	18.3	17.9	0.552
Concern about disease	50.0	51.0	47.3	0.834
Satisfaction with patient-clinician relationship	77.7	87.5	83.8	0.079
Positive beliefs	65.0	65.8	70.1	0.349
Treatment convenience	67.7	74.3	73.2	0.396
Self-declared compliance	88.2	94.8	93.7	0.213

^a^Compliance profiles defined from data collected with the Travalert device.

Patients in the low compliance profile group reported the lowest score for satisfaction with patient-clinician relationship, positive beliefs, treatment convenience and self-declared compliance dimensions (mean of 77.7, 65.0, 67.7 and 88.2, respectively), and the highest score for the concern about treatment dimension (mean of 22.2). As regards the concern about disease dimension, patients in the low compliance profile group reported a mean score close to that of patients in the moderate compliance profile group, which reported the highest score for this dimension (mean of 50.0 and 51.0, respectively).

## Discussion

The EDSQ was developed to measure satisfaction and compliance of patients with glaucoma treatment. The aim of the present study was to create the scoring method and to assess the psychometric properties of the EDSQ so that the questionnaire could be used in future studies.

The questionnaire was well accepted by patients, as shown by a good quality of completion of the EDSQ. The psychometric properties of the hypothesized structure of the EDSQ, which included five dimensions, were unsatisfactory, with a weak internal consistency reliability of most dimensions and an overall poor construct-related validity. Thus, it was decided to define an alternative structure using a PCA that grouped the items into eight factors. For two of the eight factors, no single underlying concept was observed between the items grouped into each of these two factors; they were therefore not included in the structure. For the six other factors, a single underlying concept was identified between the items grouped into each of these six factors: five items related to concern about treatment; two items related to concern about disease; three items related to positive beliefs; five items related to satisfaction with patient-clinician relationship; three items related to self-declared compliance; and three items related to treatment convenience. A score was then calculated for each of the six final dimensions. Some final dimensions were close to the hypothesized ones, such as the satisfaction with patient-clinician relationship dimension with the patient-clinician relationships hypothesized dimension; the concern about treatment dimension with the apprehension hypothesized subdimension; and the patient-treatment relationship dimension with the treatment convenience hypothesized dimension. The major difference between final and hypothesized dimensions was observed for the items of the patient experience hypothesized dimension that were scattered over several dimensions in the final structure. The six-dimension structure defined using the PCA presented a good construct-related validity, as well as a satisfactory internal consistency reliability. In addition, patients younger than 60 years reported more concerns about disease compared with older patients, as well as women compared with men, which could have been expected as a similar relationship of disease-related concerns with age and gender was already observed in several diseases such as cancer[19,20], bowel disease[21] or diabetes[22]. The 43-item version of the EDSQ used in our study was slightly modified while our study was being carried out, i.e. the wording was modified for seven items, one item was divided into two distinct items, the response scale was modified for one item and two items, which are not included in the final scoring presented in this paper, were removed. Although these modifications may result in minor changes in future studies compared with our results, they were made to improve the content of the questionnaire and can be expected to improve the psychometric performances of the EDSQ.

A ceiling effect was observed for the satisfaction with patient-clinician relationship and the self-declared compliance dimensions as 21% and 49%, respectively, of patients reported the highest possible score, resulting from a skewed distribution for items related to patients' satisfaction and self-declared compliance. However, those results are not surprising as acquiescent response bias[23-25], which is the tendency to agree with items rather than disagree, and social desirability bias[26], which is the will to give a positive self-image to the physician, are well-known phenomena when measuring patient satisfaction and self-declared compliance.

According to theoretical satisfaction models and results previously reported in the literature, a relationship between satisfaction and compliance was expected. Even if no statistically significant relationship was demonstrated in our results, a clear pattern appeared in the distribution of EDSQ dimension scores among compliance profile groups defined using Travalert data. Even though the probability to observe this result was very low, patients in the low compliance profile group reported the most unfavourable score compared with the moderate and high compliance profile groups for five of the six EDSQ dimensions. This result indicated a higher level of concern about treatment, and a lower level of satisfaction with patient-clinician relationship, treatment convenience, positive beliefs and self-declared compliance for those patients than for patients considered as moderately or highly compliant. Lastly, the association between satisfaction and compliance appeared more complex and was reported elsewhere[27].

We acknowledge that our study had some limitations. The version of the EDSQ we used is not the final one as the questionnaire was slightly modified while our study was being conducted, and our sample size was relatively limited to enable the generalization of our results. It would thus be necessary to confirm the final structure presented in this paper using the final version of the EDSQ and a larger sample size. The cross-sectional design of this study did not allow the longitudinal psychometric properties of the questionnaire, including test-retest reliability and responsiveness to change over time, to be assessed. Future longitudinal studies should therefore be conducted to enable these properties to be evaluated. Such studies would also enable the dynamic process linking satisfaction to compliance to be explored. Travalert is limited to Travatan^® ^and DuoTrav^® ^and therefore extrapolation to other products would need additional data collection. Finally, the association between clinical characteristics, such as the severity of visual loss and length of time with glaucoma therapy, and satisfaction and compliance was not analysed but could be an interesting point to focus on in future studies.

Measuring patients' compliance is a complex problem as many methods exist to determine how well patients follow physicians' instructions regarding a treatment regimen[28,29]. Subjective measures, such as the EDSQ self-declared compliance dimension score in our study, tend to cost less than objective measures, but also to overestimate compliance. This is partly due to the social desirability bias, which is consistent with the ceiling effect observed for the EDSQ self-declared compliance score in our study. Objective measures such as the data collected with the Travalert electronic device, which were used to define three compliance profiles in our study, can monitor the number of medications taken as well as the times that medications are taken. However, these methods tend to overestimate compliance as patients can use the device without actually taking the medication. Both subjective and objective measures have advantages and disadvantages, and no one measure has proved to be better than the others. No gold standard has been established for the assessment of compliance[28,29], and therefore comparing two methods remains difficult. However, the EDSQ self-declared compliance score has been shown to be in line with the compliance profiles defined using data collected with the Travalert electronic device, which is encouraging as the power of analyses was limited. These findings indicate that alongside objective measures, the EDSQ could be used in more complex and possibly prospective studies to comprehensively describe the relationships between patients' perception of the disease and its treatment, their satisfaction with the treatment, their self-declared compliance and the treatment effects.

## Conclusions

This study allowed a scoring method for the EDSQ to be defined and satisfactory psychometric properties of the questionnaire to be demonstrated. The EDSQ is a potentially valid and reliable instrument that could be used in future studies to assess patients' satisfaction and compliance with their eye-drop treatment for glaucoma or IOP.

## Competing interests

Antoine Regnault, Muriel Viala-Danten and Hélène Gilet are paid consultants to AlconLabs. Gilles Berdeaux is an employee of AlconLabs.

## Authors' contributions

AR designed and programmed statistical analyses, participated in the interpretation of results and helped draft the manuscript. MVD participated in the design of statistical analyses and the interpretation of results. HG participated in the interpretation of results and drafted the manuscript. GB conceived the study and participated in study design and interpretation of results. All authors read and approved the final manuscript.

## Copyrights

EDSQ is protected by copyright with all rights reserved to ALCON. Do not use without permission.

## Pre-publication history

The pre-publication history for this paper can be accessed here:

http://www.biomedcentral.com/1471-2415/10/1/prepub
